# Electronic reporting of patient-reported outcomes in a fragile and comorbid population during cancer therapy – a feasibility study

**DOI:** 10.1186/s12955-020-01480-3

**Published:** 2020-07-11

**Authors:** Gry Assam Taarnhøj, Henriette Lindberg, Line Hammer Dohn, Lise Høj Omland, Niels Henrik Hjøllund, Christoffer Johansen, Helle Pappot

**Affiliations:** 1 Department of Oncology, University of Copenhagen, Rigshospitalet, Blegdamsvej 9, 2100 Copenhagen Ø, Denmark; 2 Department of Oncology, University of Copenhagen, Herlev Hospital, Herlev, Denmark; 3 AmbuFlex/WestChronic, Occupational Medicine, University Research Clinic, Aarhus University, Herning, Denmark; 4 Department of Clinical Epidemiology, Aarhus University Hospital, Aarhus, Denmark

**Keywords:** Patient-reported outcomes, ePRO, Bladder cancer, Chemotherapy, Immunotherapy, Feasibility

## Abstract

**Background:**

Electronic collection of patient-reported outcomes (ePROs) is becoming widespread in health care, but the implementation into routine cancer care during therapy remains to be seen. Especially, little is known of the use and success of electronic reporting during active cancer treatment in fragile and comorbid patients. The aim of this study was to test the feasibility of ePRO and its incorporation into routine cancer care, measured by physician compliance, for a fragile and comorbid bladder cancer (BC) population receiving chemo- or immunotherapy.

**Methods:**

All BC patients initiating treatment for locally advanced or metastatic bladder cancer at Rigshospitalet or Herlev Hospital, Denmark, were approached during an 8 month period. Exclusion criteria were patients not speaking Danish or not being signed up for electronic communication with health authorities. Enrolled patients were prompted to complete weekly ePROs from home. Patients completed the European Organisation for Research and Treatment of Cancer’s general quality of life questionnaire, QLQ-C30, and the module for muscle-invasive bladder cancer QLQ-BLM30, the Hospital Anxiety and Depression Scale, HADS, and selected items from the Patient Reported-Outcomes version of the Common Terminology Criteria of Adverse Events (PRO-CTCAE), in total 158 questions weekly. If failing to report when prompted, patients were sent two e-mail reminders. Patients were informed that the physician would have an overview of the reported ePROs at their following clinical visits. Physicians were at all clinical visits informed to look at the ePROs in a software solution separate from the medical records. Physicians were logged to check their compliance to the task. No continuous surveillance of ePROs was established.

**Results:**

Of 91 patients screened for enrolment, 19 patients (21%) were not found eligible for standard treatment, eight patients (9%) were not signed up for electronic communication with the health authorities and nine patients (10%) declined participation. Another six patients did not meet other inclusion criteria. In total 49 BC patients were enrolled, 29 initiating chemotherapy and 20 initiating immunotherapy. A total of 466 electronic questionnaires were completed. The overall adherence of the patients to complete ePROs was at an expected level for an elderly cancer population (75%) and remained above 70% until the 6th cycle of treatment. The physician’ compliance was in contrast low (0–52%) throughout the course of treatment.

**Conclusions:**

Electronic reporting of PROs is feasible in a fragile and comorbid population of patients during routine active cancer treatment. Despite clear implementation strategies the physician compliance remained low throughout the study proving the need for further implementation strategies.

## Background

In previous studies we have seen that bladder cancer patients have very poor clinical outcomes with 66% of the patients experiencing hospitalisation during oncological treatment and only 45–50% of patients completing the planned treatment, whether as neoadjuvant treatment or treatment for metastatic disease [1–3]. One of the explanations for this could be that many of these patients deal with comorbidities troubling completion of treatment [4]. Better handling of troublesome symptoms during treatment therefore seems reasonable. The active use of patient-reported outcomes (PROs) for timely monitoring and handling of symptoms during cancer treatment has proven advantageous for cancer patients illustrated by improvements in quality of life, satisfaction with care and survival [5–9]. The improvement in survival was hypothesized to be due to better adherence to treatment [7]. Bladder cancer patients may therefore benefit from the use of electronic PROs (ePROs) as an intervention to improve adherence to treatment and hence their outcomes. However, little is known of the adherence to ePROs by specifically frail and comorbid patients as previous studies focus on a broad population of cancer patients across diseases [5, 6, 10].

To obtain improvements in clinical outcomes as a result of active use of PROs requires staff dedicated to management and action on the reported symptoms, whether reported on paper or as ePROs [11–13]. The technological developments urge the health care systems to start implementation of ePROs [14–16] and many studies report on the feasibility and implementation of ePROs in different populations [10, 17–19]. However, as pointed out in previous studies several challenges exist with ePRO implementation in many of which the clinicians play a vital role in the success of implementation [11, 20, 21]. One of the key challenges found by Nordan et al. was the acceptance of a new workflow. The handling of ePROs was, instead of a route to better and more efficient patient care, perceived as an additional daily task not part of the available clinical guidelines [11, 22]. To study this reluctance from clinicians, qualitative research studying physicians’ perceptions of ePRO has been carried out [17, 23, 24]. However, these studies do not report quantitively on the clinician use of ePROs to estimate the impact of a given implementation. Before extensive costs across health care systems are used to implement a given software for this purpose, analysis on the feasibility and real-life use of such in different populations is warranted.

Working towards the possible use of PRO in clinical practice, the purpose of this study was to test the feasibility of ePRO from a patient and clinician perspective. This study therefore reports on the adherence to ePROs in a fragile and comorbid population of bladder cancer patients in which ePROs may have a substantial potential to improve clinical outcomes. This study will also present quantitative data on physician compliance with ePRO completion and review.

## Methods

From 1 February 2018 all patients with bladder cancer initiating standard treatment with chemotherapy or immunotherapy outside clinical trials at the Departments of Oncology at University Hospitals of Copenhagen, Rigshospitalet and Herlev Hospital, Denmark, were approached. Patients fulfilling the following inclusion criteria were asked to participate:

- Initiating standard treatment with chemotherapy (combination cisplatin-gemcitabine, carboplatin-gemcitabine or single agent vinflunine) or immunotherapy (pembrolizumab).
- Able to read Danish.
- No serious cognitive impairment as evaluated by the treating team of physician and/or nurse.
- Access to electronic communication with health authorities (e-Boks – an electronic, closed two-code guarded system, see below) [25].

Inclusion was planned until a minimum of 40 patients (minimum 20 patients initiating chemotherapy and minimum 20 patients initiating immunotherapy), were enrolled. The sample size was planned according to recommendations for pilot studies [26, 27].

The electronic system for patient communication with authorities and companies, e-Boks, is a national system based on the civil registration number unique to every Danish citizen. More than 95% of the Danish population are active in the e-Boks communication system that ensures delivery of official communication from Danish authorities and companies regardless geographical home address. Exemption from electronic communication with the authorities can be granted due to physical or mental incapacity disabling electronic communication [28]. Access to documents can only be achieved by a two-factor log-in system with two different codes unique to the citizen. Ambuflex, a generic web-based PRO system, was used as the ePRO software system and generated e-mails to the e-Boks system with links to the questionnaires [29]. Originally developed in 2004 by author NHH the Ambuflex software platform has since been implemented for out-patient follow-up for 35 diagnosis groups across 10 hospitals in Denmark [20, 30]. If enrolled patients did not have access to a computer, tablet or smartphone at home, a tablet with free data access was provided from the department during the study period.

**Fig. 1:**
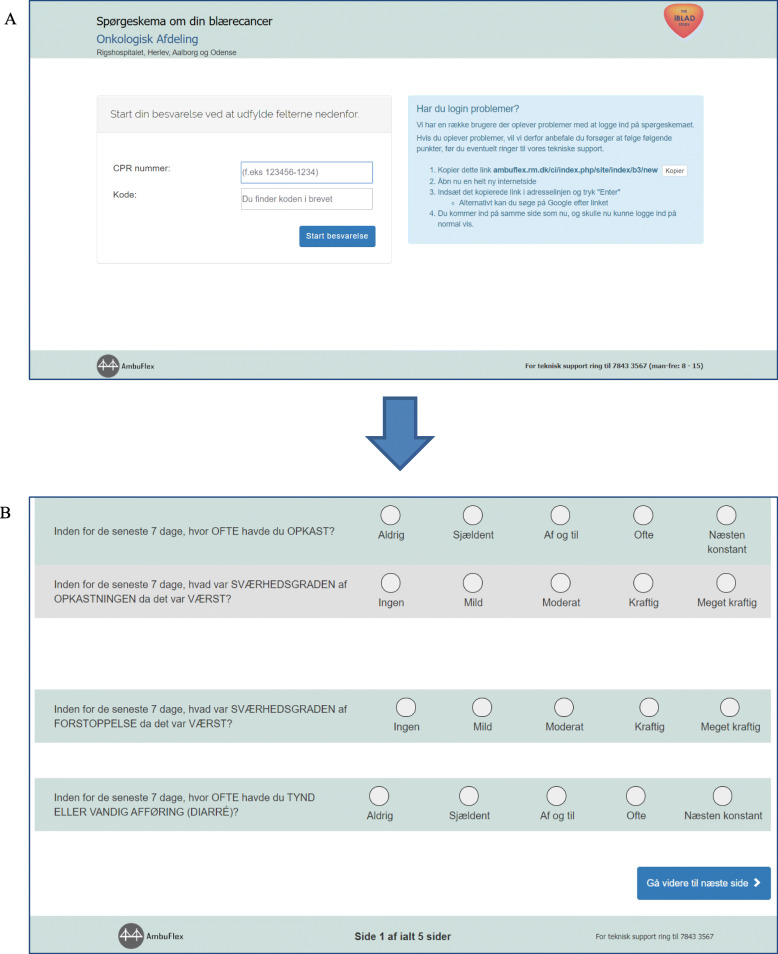
Patient view of log-on procedure and questionnaire. Shows the screen view for patients when logging into the system (**a**) with the study logo (iBLAD) in the top right corner and when completing questionnaires (**b**). The current and total number of screens is listed at the bottom of the questionnaire, so the patients are aware of their progress. Approximately 10 screens had to be completed in order to complete the full questionnaire, estimated duration 15–30 min depending on pre-existing computer skills. The Ambuflex telephone support number is listed at the bottom in case of difficulty logging in or when completing the questionnaire

All patients completed the following questionnaires weekly electronically through e-Boks: European Organisation for Research and Treatment of Cancer (EORTC) core quality of life questionnaire: QLQ-C30 [31], EORTC module for muscle-invasive bladder cancer: QLQ-BLM30 [32], the Hospital Anxiety and Depression Scale (HADS) [33] and selected Patient-Reported Outcome version of the Common Terminology Criteria for Adverse Events (PRO-CTCAE) questions [34]; a total of 158 questions by each patient throughout the course of treatment. All questionnaires had a recall period of 1 week. The QLQ-C30 consists of 30 questions for assessing quality of life in cancer patients and comprises nine multi-item scales; five functional scales, three symptom scales and a global health scale [31]. It has been validated and translated into more than 100 languages, including Danish [35]. The bladder cancer specific module for muscle-invasive disease, QLQ-BLM30, can be used as a supplement to the QLQ-C30. It consists of 30 questions, divided into items concerning e.g. urinary, bowel and sexual functions [36]. The HADS is a brief but commonly used scale [33, 37]. The scale consists of 14 questions, divided into two subscales for anxiety and depression, all items rated on a 4-point scale. The PRO-CTCAE library was developed by the National Cancer Institute, US, in 2014 [34]. It consists of 124 items representing 78 symptomatic toxicities from which the relevant items are chosen depending on the study population. The PRO-CTCAE was translated into Danish in 2016 [38]. Responses are provided on a 5-point Likert scale (0–4). The selected 45 PRO-CTCAE symptoms explored by 84 PRO-CTCAE single items were chosen as part of an item selection process described in detail in a separate paper [39]. As all participating patients had existing access to e-Boks, no specific training in opening the questionnaire was found necessary. Reminders to complete questionnaires were sent one and 2 days after the weekly questionnaire was sent. The first questionnaire was sent on the day of initiating treatment. A screen shot of the login procedure and questionnaire friendliness is provided in Fig. 1. The estimated time for completion by the patients was 15–30 min, depending on pre-existing computer skills.

Before study initiation, an implementation process for the clinical staff was undertaken. The process consisted of several initiatives: 1) Collective teaching for physicians and nurses about the purpose of the ePROs for this patient group and how to access the electronic module for viewing of the patients’ ePROs. These instructions were repeated twice within 2 months prior to enrolment start. 2) Instructions on how to access the ePRO system were also reviewed one-to-one for all physicians with out-patient duties in both uro-oncological out-patient clinics and 3) instruction cards were present and visible in all consultation rooms. Finally, 4) every patient visit in the ambulatory programme in the electronic health record system was marked with the study name (iBLAD) with a reminder to check the patient’s symptoms in the Ambuflex software. 5) The study investigator (GAT) furthermore provided everyday telephone hot-line service assistance, if needed.

For the physicians, access to the completed ePROs was through a browser-based existing clinical system that until recently was used for chemotherapy prescription, therefore well-known to all regular personnel. The estimated added workflow in opening and viewing the ePROs through this system was estimated to 2–5 min per patient per visit.

Patient adherence was measured as number of completed PRO-questionnaires divided by the number of PRO-questionnaires sent out during treatment. Physician ePRO views were logged and physician compliance was measured by number of ePROs opened in relation to a clinical visit for each patient; e.g. if 20 out of 30 patients had their ePROs viewed in relation to a given out-patient visit, the compliance would be 67%. The study took place at two university hospitals and patients were therefore seen by specialists in uro-oncology or fellow uro-oncologists in training at each clinical visit, every third week, in concordance with the length of all treatment cycles for standard treatment with chemo- or immunotherapy.

**Fig. 2:**
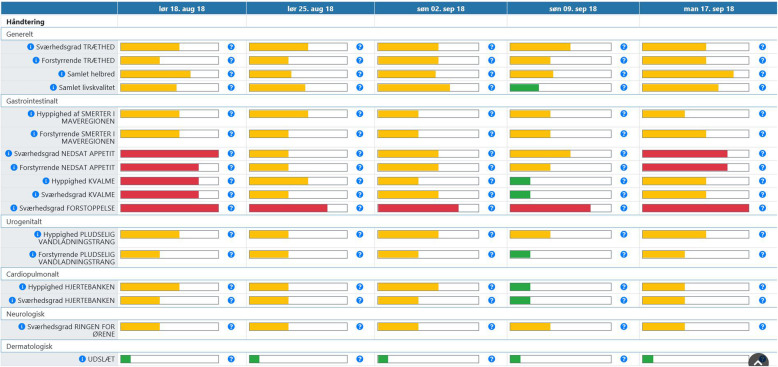
Physician view of completed PROs in the Ambuflex system. Figure 1 shows the development of symptoms (left column) over time with dates at the top. The coloured bars (green, yellow, red) indicate severity of the reported PRO-CTCAE symptom or QLQ-C30 item. In this viewing only selected symptoms chosen by the study team (physicians) are shown. The selected QLQ-C30 or PRO-CTCAE items (F: frequency, S: severity, I: interference, P: presence) are in order of appearance: Fatigue (S), fatigue (I), global health (QLQ-C30, q29), global quality of life (QLQ-C30, q30), abdominal pain (F), abdominal pain (I), decreased appetite (S), decreased appetite (I), nausea (F), nausea (S), constipation (S), urinary urge (F), urinary urge (I), heart palpitations (F), heart palpitations (S), ringing in ears (S) and rash (P). By pressing ‘Vis besvarelse’(English: show completed questionnaire) the physician can access all responses to the 158 single items

No surveillance or alert system was provided for the ePRO system, thus no alerts to patients nor clinic were provided while patients reported from home. The ePRO system therefore represented a passive collection of data from the patient with the possibility to incorporate these data into the out-patient consultation between physician and patient e.g. to improve physician-patient communication about symptoms during treatment or as an overview for the physician, Fig. 2. Eleven symptoms were chosen as the ‘front page’ in the ePRO system by two participating physicians, Fig. 2. All other responses could be seen by one additional click on the screen. All symptoms were marked with either green, yellow or red, depending on severity of the symptom, thereby enabling quick overview of troublesome symptoms.

The study was approved by The National Data Protection Agency *(file no.: 2012-58-0004).* All participants completed written informed consent in accordance with Danish ethical legislation. Patients could according to the Helsinki Declaration always decline further ePRO completions without providing a reason. Patients ceased ePRO completion after 6 cycles of treatment (18 weeks) or when terminating treatment, for whatever reason.

## Results

From February 2018 through September 2018, 91 patients referred to the Department of Oncology at Rigshospitalet or Herlev Hospital were screened for study enrolment 19 patients (21%) did not meet the prespecified inclusion criteria of initiating standard treatment either because of poor performance status preventing treatment initiation or due to inclusion into a clinical trial, eight patients (9%) did not have access to electronic communication, three patients (3%) declined treatment, three patients (3%) were missed at treatment initiation and nine patients (10%) declined participation, the majority due to reluctancy with information technology (IT) or not wanting to spend time completing questionnaires. The inclusion rate for patients initiating chemotherapy was higher than for immunotherapy and therefore study inclusion continued until 20 patients initiating immunotherapy were enrolled, thereby enrolling 29 patients initiating chemotherapy and 20 patients initiating immunotherapy.

Demographic data for participants is shown in Table 1. The distribution between patients starting neoadjuvant treatment vs. treatment for metastatic disease was 27 and 73%, respectively. Fifty-five percent of patients did not complete the planned treatment. The main reasons for early treatment cessation were disease progression (48%), death (11%), nephrological toxicity (7%), haematological toxicity (7%) or infection (7%).

**Table 1:** Patient characteristics

	*N* = 49 (%)
Age (median(range))	68 (35–82)
Gender	
Female	7 (14%)
Male	42 (86%)
Disease	
Locally advanced	13 (27%)
Metastatic	36 (73%)
Treatment	
Cisplatin-gemcitabine	24 (49%)
Carboplatin-gemcitabine	3 (6%)
Pembrolizumab	20 (41%)
Vinflunine	2 (4%)
Completed cycles (median(range))	4 (1–6)
Completion of planned treatment	
Yes	22 (45%)
No	27 (55%)
Hospital admission during treatment	
Yes	27 (55%)
No	22 (45%)

A total of 466/624 (75%) electronic questionnaires were completed by the patients. Baseline patient ePRO completion was achieved for 94%. The patient adherence remained above 70% throughout the 6 cycles of chemo- or immunotherapy only to drop after the sixth cycle of treatment. The completeness of data across all questionnaire items was 97% and in 76% of all questionnaires all 158 items were completed. Two patients withdrew consent to complete questionnaires during the study due to IT-problems with at-home completion but did not withdraw consent to participation in the study.

A total of 163 physician view logs were registered throughout the study. Multiple logs from the same physician on the same day for the same patient and logs by author GAT as principal investigator were filtered out leaving 40 physician view logs from a total of 24 different physicians participating at any given time during the course of the study. None of the other authors of this paper had log views to filter out and none of the authors had access to log-data while the study was being conducted.

**Fig. 3:**
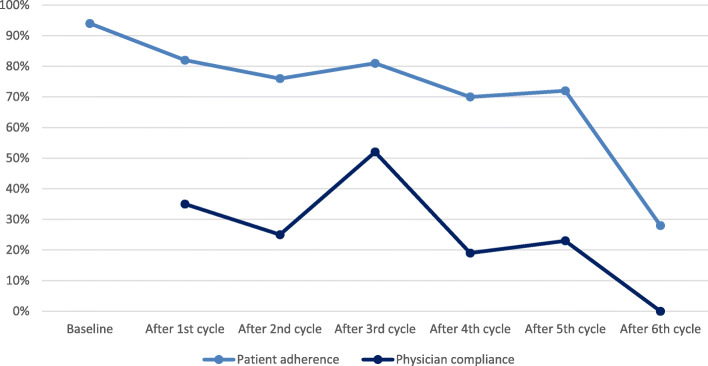
Patient adherence and physician compliance with ePRO completion and views. The curve for patients indicates the percentage of questionnaires completed for patients still receiving treatment. The curve for physicians indicates the percentage of physicians viewing a completed questionnaire in relation to an out-patient visit

The 49 patients completed a total of 208 cycles of treatment and thus had a total of 208 visits at the hospital at which the physician could view the patients’ responses. However, as patients adherence was not 100% only 128 clinical visits had a completed questionnaire in conjunction with the visit, see total number of completed questionnaires in Table 2. Each log was checked against the electronic health record (EHR). If the log view was in relation to a contact with the given patient the log counted as viewed in the final count. The amount of physician log views dispersed by outpatient visits are displayed in Table 2 and Fig. 3. The physician log showed a compliance with the ePRO system of 35% of questionnaires viewed at first outpatient consultation after initiating treatment. Physician compliance remained considerably lower (0–52%) than that of the patients throughout the course of the patients’ treatment.

**Table 2:** Patient adherence and clinician view of ePRO completion

Patient adherence	Number of completed questionnaires	Number of questionnaires sent out	%
Baseline	46	49	94
After 1st cycle	37	45	82
After 2nd cycle	32	42	76
After 3rd cycle	25	31	81
After 4th cycle	16	23	70
After 5th cycle	13	18	72
After 6th cycle	5	18	28
Physician views	Number of viewed questionnaires	Number of completed questionnaires	
Baseline^a^	N/A	46	–
After 1st cycle	13	37	35
After 2nd cycle	8	32	25
After 3rd cycle	13	25	52
After 4th cycle	3	16	19
After 5th cycle	3	13	23
After 6th cycle	0	5	0

^a^ The baseline questionnaires were seen by the clinician at first consultation after initiating treatment, e.g. after 1st cycle of treatment

## Discussion

This study gives real-life insight into the implementation of ePROs in daily cancer care. Our data showed high patient adherence (70–94%) with ePRO completion throughout the 6 cycles of oncological therapy in an elderly and comorbid patient population while physician compliance was considerably lower (0–52%) despite a clear strategy for implementation. The results of this study are important to better understand the complexity of ePRO implementation and create strategies for further application.

The patient adherence with ePRO completion found in this study is similar to the findings in previous comparable studies examining ePRO adherence [10, 19, 40]. The drop seen in patient adherence after 6 cycles of chemo- or immunotherapy treatment may likely be due to the passive nature of the ePRO collection not creating alerts to neither patient nor clinic but simply gathering of data from the patient. Also, lack of feedback from the ePRO completion at clinical visits through the low clinician compliance seen in this study is known to make patient engagement drop [23].

Possible explanations for the low clinician compliance may be many. First, although the physicians were reminded before every enrolled patient and proposed to do so by the team management, the viewing of ePROs by physicians was not deemed mandatory by department management. As pointed out by Nordan et al. this may have affected physician adherence [11]. Second, the ePRO reporting started shortly after implementation of a new EHR system, into which the ePROs were not automatically transferred thus likely to be reflected in adherence by physicians [24]. Clinicians were likely reluctant to engage in additional electronic systems for care of patients due to the busy workflow thus resulting in poor clinician adherence to the study. Also academically trained personnel, e.g. physicians, may be opposed to implementing PROs based on the still sparse literature of the benefits on QoL and survival, two central elements in clinical oncology. Finally, the study took place at two large university hospitals in which specialist training and multiple clinical trials take place. Many oncologist residents and fellows not permanently allocated to the hospital or department therefore take part in the daily out-patient clinics perhaps disturbing continuity of permanent staff or not being fully introduced to the purpose of this study. This could have affected clinician adherence to view of the ePROs [11, 24].

This study meets the recommendations for successful implementation as depicted by Snyder et al. in the ISOQOL User’s Guide for Implementing Patient-Reported Outcomes Assessment in Clinical Practice [13] and the majority of the criteria related to successful implementation by Nordan et al. but may have come short on two issues despite the multifaceted strategy for implementation: 1) engaging providers and staff in a new workflow and 2) overcoming the EHR data management issues when the ePROs are not merged into the EHR. The turning point for Nordan et al. had been engaging a “physician champion” to engage colleagues by demonstrating the flexibility of the tool and highlighting efficiencies in workflows [11]. While this may be an important factor to build future learnings upon, one may also hypothesize that more quantitative research informing the physicians of e.g. time savings or similar efficiency pathways in the care of patients when using ePROs could be an important factor. Especially as the time saved not having to go through checklists of symptoms in the physician’s office may be spent worthwhile with the patients thus improving satisfaction with the quality of care delivered [22]. Gaining a “physician champion” does not however compensate the need for management endorsement of ePROs as part of daily clinic. This issue may be overcome through implementation of ePRO into the clinical guidelines thus endorsing the clinical advantage with the use of ePROs [24].

Finally, despite intentions to test the feasibility of ePROs in a frail and comorbid population of patients with high rates of hospitalisations and treatment cessations this study is limited by the inclusion criteria necessitating existing access to electronic communication with authorities through e-Boks. This inclusion criteria ruled out 9% of possible study participants, most likely the 9% most frail patients as exception from e-Boks communication is only allowed in certain circumstances (substantial morbidity or disability) after approval from the authorities [28]. Future work on how to include these frail patients into similar studies is needed. Also, this study may have had better success in terms of high physician compliance through a more in-depth training of personnel or by restricting clinical consultations with the task of ePRO viewing in the current study to only a few physicians. However, these changes would require more allocated time away from clinic for a more thorough onboarding task and also a re-design of clinical duties for attending staff in the university hospitals which was not the objective of this study.

This fragile and comorbid population is in need of interventions to better their outcomes [1]. The results of this study have contributed to the planning of a national randomized trial in the bladder cancer population in which ePROs act as the intervention for timely symptom management (NCT03584659). The randomized study has incorporated the learnings of the current study e.g. by increasing patient involvement through alerts in real-time for patients at home instead of alerts to clinic thus minimizing the added ‘burden’ for physicians. The results will be reported following completion of enrolment, in the autumn 2020.

## Conclusions

This study gives real-life insight into the implementation of ePROs in cancer care and highlights that physicians may need more quantitative documentation of the positive benefits of using ePROs in order to endorse the use of ePROs in daily clinic. Thus, this study urges for continuous feasibility testing before software implementation, at the very least in the respect of both patients and providers.

## Data Availability

The data that support the findings of this study are available from corresponding author Gry Assam Taarnhøj but restrictions apply to the availability of these data, which were used under license for the current study, and so are not publicly available.

## Abbreviations

ePROs: Electronic patient-reported outcomes
EORTC: European Organisation for Research and Treatment of Cancer
QLQ-C30: EORTC’s general core quality of life questionnaire
QLQ-BLM30: EORTC’s module for muscle-invasive bladder cancer
HADS: Hospital Anxiety and Depression Scale
PRO-CTCAE: Patient-Reported Outcomes version of the Common Terminology Criteria for Adverse Events
PRO(s): Patient-reported outcome(s)
EHR: Electronic health record
ISOQOL: International Society of Quality of Life
